# Influence of Psychological Need-Based Teachers’ Autonomy Support on Effectiveness and Engagement in English Learning

**DOI:** 10.3389/fpsyg.2021.663374

**Published:** 2021-07-02

**Authors:** Pengkai Liu

**Affiliations:** School of Foreign Languages, Northwest University of Political Science and Law, Xi’an, China

**Keywords:** psychological needs, teachers’ autonomy support, students in secondary school, English, effectiveness, engagement

## Abstract

It is to explore the effects of psychological needs and teachers’ autonomy support on students’ English learning, hoping to provide a realistic basis for the innovation of English teaching. Four hypotheses are proposed first, and 420 students are then randomly selected from seven classes in Xi’an Ai Zhi Zhong Xue in Shaanxi Province as the research subjects, with 420 questionnaires distributed. After invalid questionnaires are excluded, a total of 400 valid questionnaires are collected. Then, SPSS26.0 is employed to analyze the collected scale data. The average value of emotional exhaustion and the average value of low sense of achievement are relatively close in the direction of English learning burnout, which are larger than that in the other two directions. The three directions in English learning burnout are positively correlated with each other. The three directions in English learning engagement are positively correlated with those of psychological needs. Besides, there is an obvious positive correlation between the three directions of English learning engagement with teachers’ autonomy support (*p* < 0.01). However, the three directions in teachers’ autonomy support and the three directions in psychological needs are all negatively correlated with English learning burnout (*p* < 0.01). To sum up, the psychological needs of middle school students are effectively met, and teachers’ autonomy support can obviously promote the effectiveness and engagement of middle school students’ English learning.

## Introduction

Teachers’ autonomy support means that students can feel the teacher’s support for their own choices and decisions, and can get meaningful information and appreciation from the teacher, thereby alleviating stress (Anderhag et al., 2015). The connotation of teachers’ autonomy support not only pays attention to the three psychological needs of the individual development, but also attaches great importance to the improvement of the school education environment (Saeki and Quirk, 2015), thereby connecting students’ inner needs with the external environment. Students’ learning initiative is enhanced with teachers’ autonomy support. Students take the initiative to manage their own learning process while being responsible for their own behavior (Chin et al., 2015). Furthermore, it also encourages students to tap their own internal potential and evaluate themselves correctly according to their personality characteristics and hobbies (Jing and Yeung, 2016).

There are three basic psychological needs within an individual: autonomy, competency, and relatedness (Anja et al., 2016). Autonomy refers to self-determination, representing the individual’s sense of choice and sense of self in the activity. Competence indicates that an individual believes that he can reach a certain goal and that he is competent for the prescribed task. Relatedness means that individuals need care, understanding, and support from others, and long for a sense of belonging. The above three basic needs are as important to humans as the air, sunlight, and soil to plant growth, and they are indispensable to individual humans (Dehaan et al., 2015). Studies reported that meeting the basic psychological needs of individuals can enable individuals to grow up mentally healthy and gain a sense of happiness. If the individual’s psychological needs are not satisfied, the individual will have certain psychological problems (Martela and Ryan, 2016).

To meet the basic psychological needs of students, domestic and foreign researchers have conducted a lot of research on related influencing factors. Currently, there is a theory recognized worldwide, that is, teachers’ autonomy support can meet the autonomous needs of students in the learning process, and will ultimately promote students’ learning and physical and mental development (González et al., 2016; Wang et al., 2019). Feeling the support of others can enhance the happiness of individuals. Studies found that the teachers’ autonomy support helped to meet their basic psychological needs and improved the learning efficiency of students (Pope and Hall, 2015). Students under teachers’ autonomy support tend to have good academic performance. They have a strong sense of happiness and can adapt to society more quickly (Halty et al., 2019). At present, there are many theoretical and practical studies on teachers’ autonomy support in foreign countries, while there are few empirical studies related to teachers’ autonomy support domestically. What is more, the connotation of teachers’ autonomy support is grounded in self-determination theory, which is a cultural product of the Western world (Marsh et al., 2015; Ewing et al., 2016). Whether this theory can be applied to the actual environment in China requires in-depth research.

To study clearly whether teachers’ autonomy support has an impact on students’ English learning, based on relevant theories, the middle school students of Xi’an Ai Zhi Zhong Xue in Shaanxi Province are selected as research subjects. Questionnaires are distributed to understand the English learning process of middle school students, so as to analyze the effects of psychological needs and teachers’ autonomy support on the effectiveness and engagement in English learning. It is expected to provide empirical evidence for the innovative English teaching methods.

## Materials and Methods

### Establishment of Hypotheses

Teachers’ autonomy support means that teachers think from the perspective of students, reduce compulsive behaviors on students, care about students’ learning emotions, and show support for students’ behaviors. With teachers’ autonomy support, students are happy and relaxed because they can feel the teacher’s support and care. At the same time, they will have great learning motivation during the learning process. A teacher with a high degree of autonomy support actively stimulates and cultivates students’ interest in learning according to the laws of students’ physical and psychological development, enables students to make different choices during the learning process, and allows them to express themselves. A teacher with autonomy support appreciates students, supports their motivations, and understands their behaviors. Specifically, he/she is able to accept students’ different perspectives, and accept students’ negative emotions and give them timely understanding and comfort. In this study, “teachers’ autonomy support” refers to the degree of support that teachers show for students.

Some researchers pointed out that the teachers’ autonomy support can stimulate students’ learning initiative, so that they learn actively and are responsible for themselves. In addition, teachers’ autonomy support is conducive for individuals discovering their own personality characteristics and interests, so that they can build their own evaluation system. The junior high school time is an important growth period. With the physiological changes, as well as the further development of cognitive ability, junior high school students begin to understand themselves deeply and systematically, forming a complete personality in the interaction with the environment (Chen, 2019). Environmental factors are important factors affecting the academic performance of junior high school students, and parents and teachers are the main influencing factors in the process of student growth. It is necessary to pay long-term attention to the study and life of junior high school students. The satisfaction of psychological needs and teachers’ autonomy support will affect students’ English learning burnout. However, what is the relationship between psychological need satisfaction and teachers’ autonomy support? Is there a mediating effect? Based on this, the following four hypotheses are proposed for analysis.

First: Students will experience burnout in English learning. Second: Teachers’ autonomy support has an important impact on students’ English learning burnout. They are negatively correlated with each other. Third: Psychological needs have an effect on students’ English learning burnout. Fourth: Teachers’ autonomy support influences students’ English learning burnout. Furthermore, it can affect students’ burnout in English learning through the mediating effect of satisfying psychological needs.

### Research Subjects

In the study, with the English learning of junior high school students as the research direction, 420 students (250 boys and 170 girls) from seven classes in the second grade of Xi’an Ai Zhi Zhong Xue in Shaanxi Province are randomly selected as research subjects, with 420 questionnaires distributed. Five trained researchers guide the students to finish the questionnaire. After invalid questionnaires are excluded, a total of 400 questionnaires are left, and the effective recovery rate is 95.2%.

### Research Tools

English learning burnout is a kind of negative emotion generated in the process of English learning. The actual test version of the English learning burnout scale compiled by Dr. Tao Yang (Southwest University) is adopted for analysis. The scale contains a total of three dimensions (emotional exhaustion, indifferent attitude, and low sense of achievement), with a total of 18 items. Subjects are scored according to a 6-grade scale (Table 1).

**Table 1:** The structure of English learning burnout scale.

Dimension	Number of items	Score
Emotional exhaustion	5	1–6 means never, seldom, sometimes, usually, frequently, and very frequently
Indifferent attitude	7	
Low sense of achievement	6	

The discrimination, reliability, and validity of the English learning burnout scale are relatively high, as shown in Table 2.

**Table 2:** Feasibility verification of English learning burnout scale.

Items	Tested numbers	Results
Item discrimination	Correlation coefficient = 0.361	High discrimination
Validness	Load > 3	High validness
Reliability	Cronbach *α* coefficient = 0.891	High reliability

The scale can measure the actual situation of English learning burnout for beginners in secondary school.

The basic psychological need satisfaction scale (compiled by Sheldon et al.) is applied to measure the satisfaction of the psychological needs in the process of English learning. The scale contains three dimensions (autonomy, competence, and relatedness), with a total of nine items. The average of the scores of all the questions is taken as the final score, and a higher score means a higher satisfaction degree. The load coefficient of the table is more than 0.3, and the score and Cronbach *α* coefficient of each dimension are shown in Table 3.

**Table 3:** The structure of basic psychological need satisfaction scale.

Dimension	Number of items	Cronbach *α* coefficient	Score
Satisfaction of autonomy	3	0.71	7-grade scoring
Satisfaction of competence	3	0.85	1 = total disagreement
Satisfaction of relatedness	3	0.79	2 = total agreement

Teachers’ autonomy support scale includes nine teaching behaviors as observation indicators. Two un-informed personnel are trained to be observers at the same time. They observe the teacher’s behavior in the classroom, and the frequency of the teacher’s autonomy support behavior is recorded for scoring. The structure of the scale is shown in Table 4.

**Table 4:** The structure of teachers’ autonomy support scale.

Teachers’ autonomy support behavior	Grading for times	Score
Listening to the students	1 = never	The mean value of the 9 items is taken, and higher score means more frequent autonomy support behavior of teachers
Allowing students to learn independently	2 = seldom	
Sparing time for discussion	3 = sometimes	
Explaining the basic principles to students	4 = usually	
Feedbacking	5 = frequently	
Encouraging students to answer questions		
Giving some hints		
Solving students’ confusions		
Recognizing the ideas of students		

At first, the consistency reliability of the two observers is *α* = 0.69, which is relatively low. Then, the two discuss the inconsistencies. After they reach an agreement, the consistency reliability of the two observers is *α* = 0.97. The mean value of the results by the two is the final result for the evaluation of teachers’ behavior.

The student learning engagement scale is adopted to measure the student’s English learning engagement. The scale contains three dimensions, with a total of 16 items. The scores of all 16 items are added together. The load coefficient of the table is more than 0.3, and a higher total score suggests higher engagement in learning (Table 5).

**Table 5:** The structure of learning engagement scale.

Dimension	Number of items	Cronbach *α* coefficient	Score
Behavior	5	0.87	7-grade scoring
Emotion	5	0.96	1 = total disagreement
Cognition	6	0.96	7 = total agreement

Finally, a researcher who has received relevant training saves the collected data into an excel file. The data are processed by SPSS26.0.

## Results and Discussion

### Effectiveness of English Learning of Students in Secondary School

The scores of each item in the three dimensions of the English learning burnout scale are summed up. Then, the average of the scores of each item is taken. A descriptive statistical analysis is performed on the average scores in the three directions (Table 6).

**Table 6:** English learning burnout scale scores.

Dimension	Median	Mean	Standard deviation	Skewness	Kurtosis	
Emotional exhaustion	2.21	2.02	1.09	1.02	1.65	3.19
Indifferent attitude	3.29	3.26	1.21	1.1	0.16	−0.29
Low sense of achievement	2.46	2.19	1.3	1.15	1.11	0.97

The average value of emotional exhaustion is very close to that of the low sense of achievement, both of which are less than 3 points, while that of indifferent attitude is 3.3012. The statistical results of median and mode are the same as the above characteristics. This suggests that the burnout of this overall sample is relatively low. English learning does not lead to excessive emotional exhaustion and a low sense of achievement in most subjects, and the overall learning effectiveness is relatively high. However, the subjects’ indifferent attitudes affect the learning effectiveness greatly.

### Correlation Between Teachers’ Autonomy Support With Effectiveness in English Learning

**Figure 1:**
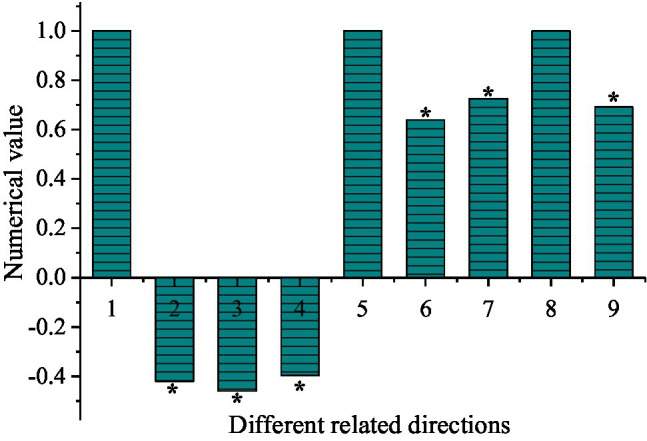
Correlation between teachers’ autonomy support with English learning burnout (^*^indicates *p* < 0.01. 1, autonomy support-autonomy support; 2, autonomy support-emotional exhaustion; 3, autonomy support-indifferent attitude; 4, autonomy support-low sense of achievement; 5, emotional exhaustion-emotional exhaustion; 6, emotional exhaustion-indifferent attitude; 7, emotional exhaustion-low sense of achievement; 8, indifferent attitude-indifferent attitude; and 9, indifferent attitude-low sense of achievement).

According to the results of the questionnaire, the relationship between teachers’ autonomy support and English learning burnout is shown in Figure 1.

The relevance score of autonomy support-autonomy support is 1, the score of autonomy support-emotional exhaustion is −0.4, the score of autonomy support-indifferent attitude is −0.45, and the score of autonomy support-low sense of achievement is −0.38, and the score of emotional exhaustion-emotional exhaustion is 1, the score for emotional exhaustion-indifferent attitude is 0.61, the score for emotional exhaustion-low sense of achievement is 0.7, the score for indifferent attitude-indifferent attitude is 1, and the score for indifferent attitude-low sense of achievement is 0.67. There is an obvious positive correlation between the three directions of English learning burnout (*p* < 0.01), but they are all negatively correlated with teachers’ autonomy support (*p* < 0.01). This indicates that teachers’ autonomy support will reduce the burnout of students in the learning process, thereby improving the effectiveness of learning.

There is an obvious negative correlation between teachers’ autonomy support and English learning burnout, which means teachers’ autonomy support can enhance the effectiveness of English learning. It is consistent with the concept that teachers’ autonomy support leads to the high effectiveness of English learning, which is in line with the results of other studies (Cervetti et al., 2015). Parents are the first object in the learning process, and the teacher is the second. Teacher’s support and harmonious teacher-student relationship can alleviate the disadaptability of students in learning English. It can also stimulate students’ sense of self-achievement in English learning, thereby enhancing their motivation to learn English (Gülbahar, 2016; Hirn, 2018). Many students fail to obtain the attention, encouragement, and support of teachers in the actual learning and become less confident in learning English. Eventually, they develop many problematic behaviors, such as not being able to complete English homework in time, and not being serious in listening and reading, which lead to English learning burnout and ultimately reduce the effectiveness of learning.

### The Relationship Between Psychological Needs and the Effectiveness of English Learning

**Figure 2:**
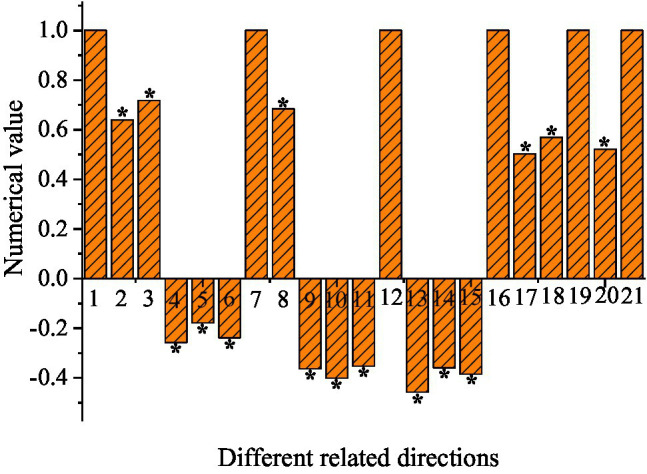
Correlation between psychological needs with English learning burnout (^*^indicates *p* < 0.01. 1, emotional burnout-emotional burnout; 2, emotional burnout-indifferent attitude; 3, emotional burnout-low sense of achievement; 4, emotional burnout-autonomy; 5, emotional burnout-competence; 6, emotional burnout-relatedness; 7, indifferent attitude-indifferent attitude; 8, indifferent attitude-low sense of achievement; 9, indifferent attitude-autonomy; 10, indifferent attitude-relatedness; 11, low sense of achievement-low sense of achievement; 12, low sense of achievement-autonomy; 13, low sense of achievement-competence; 14, low sense of achievement-relatedness; 15, autonomy-autonomy; 16, autonomy-competence; 17, autonomy-competence; 18, autonomy-relatedness; 19, competence-competence; 20, competence-relatedness; and 21, relatedness-relatedness).

Figure 2 shows the relationship between psychological needs and English learning burnout.

The scores of emotional exhaustion-emotional exhaustion, different attitude-indifferent attitude, low sense of achievement-autonomy, autonomy-competence, competition-competence, and relatedness-relatedness are all one. The scores of emotional exhaustion-indifferent attitude, emotional exhaustion-low sense of achievement, indifferent attitude-low sense of achievement, autonomy-competence, autonomy-relatedness, and competition-relatedness are all between [0.5, 0.8]. The scores of emotional exhaustion-autonomy, emotional exhaustion-competence, emotional exhaustion-relatedness, indifferent attitude-autonomy, indifferent attitude-relatedness, low sense of achievement-low sense of achievement, low sense of achievement-competence, low sense of achievement-relatedness, and autonomy-autonomy are all between [−0.5, 0], and they are all negative. The three directions of psychological needs are all positively correlated with each other (*p* < 0.01), and the three directions of English learning burnout are also positively correlated (*p* < 0.01). However, there is an obvious negative correlation between the three directions of psychological needs and those of English learning burnout (*p* < 0.01). Therefore, if the psychological needs of the students are met, the student’s English learning burnout will be reduced, that is, the satisfaction of psychological needs affect the effectiveness of English learning (Deng et al., 2021).

There is a notable negative correlation between the three directions of psychological needs and the three directions of English learning burnout. If more psychological needs are met, the degree of English learning burnout will be lower, and the effectiveness of the students’ learning will be higher. This is aligned with other related research results (Yang et al., 2015) and also consistent with the basic psychological needs theory (Liga et al., 2020). If the teacher can always pay attention to the students’ feelings and provide frequent support and encouragement to the students in the learning process, it can satisfy the students’ psychological needs in learning and create a sense of belonging in the learning process. As a result, the negative emotions of students in English learning will be alleviated, which ultimately promotes students to gain a sense of achievement, thereby improving the effectiveness of learning (Xie et al., 2018).

### Influence of Teachers’ Autonomy Support on Engagement in English Learning

**Figure 3:**
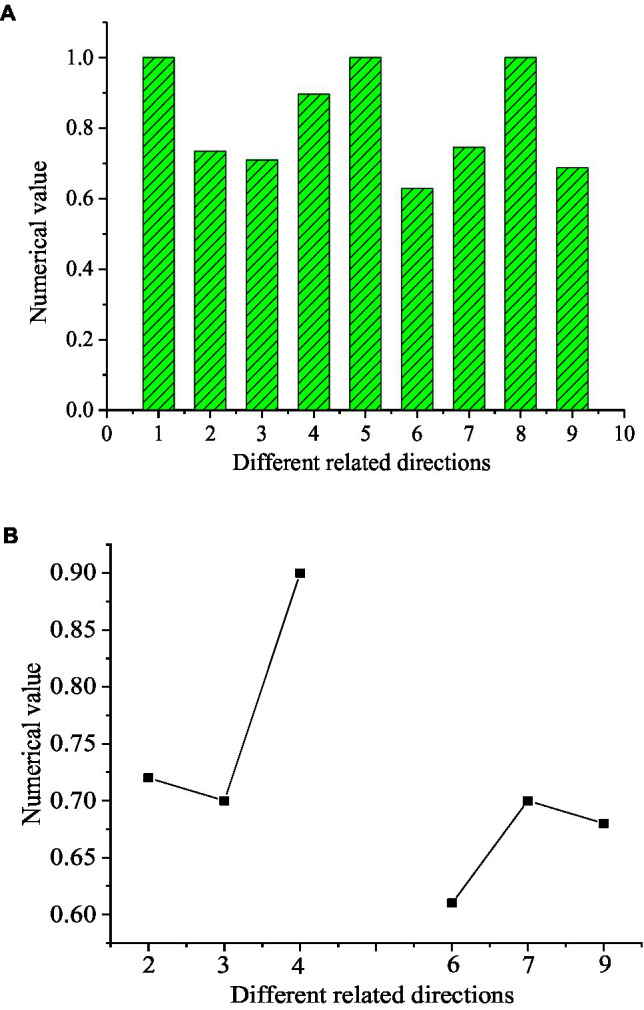
Correlation between psychological needs with engagement in English learning. **(A)** The correlation between different dimensions; **(B)** The positive correlation results. (1, autonomy support-autonomy support; 2, autonomy support-behavior engagement; 3, autonomy support-emotion engagement; 4, autonomy support-cognition engagement; 5, behavior engagement-behavior engagement; 6, behavior engagement-emotion engagement; 7, behavior engagement-cognition engagement; 8, emotion engagement-emotion engagement; and 9, emotion engagement-cognition engagement).

Figure 3 presents the relationship between teachers’ autonomy support and English learning engagement.

The scores of autonomy support-autonomy support, behavior engagement-behavior engagement, and emotional engagement-emotional engagement are all approaching 1, and the score of autonomy support-cognition engagement is 0.91. The scores of autonomy support-behavior engagement, autonomy support-emotional engagement, behavior engagement-emotional engagement, behavior engagement-cognition engagement, and emotional engagement-cognition engagement are between [0.6, 0.75]. It is evident that the three dimensions in English learning engagement are positively correlated with each other (*p* < 0.01) and at the same time, positively correlated with teachers’ autonomy support. It reports that teachers’ autonomy support will significantly enhance the engagement of students in English learning.

Teachers’ autonomy support has an obvious positive correlation with the three directions of English learning engagement, suggesting that the more teachers’ autonomy support results in higher engagement in learning, which is similar to other related research results (Lietaert et al., 2015). In the process of English learning, if students can perceive the teacher’s support, be understood, able to learn in their own way, allowed to have discussions with classmates, receive timely feedback from the teacher, or encouraged by the teacher, they will devote themselves to the English learning in a relaxed state (Bakry and Bakar, 2015; Laurenson et al., 2015).

The English learning for students in secondary school is attached great importance, it should be confirmed that students’ feelings and thought changes in the process of English learning are observed, and more care is expressed toward students. Teachers should adjust their own teaching concepts and teaching methods to meet the psychological needs of students in the process of English learning, and make students feel the understanding and support from them. This allows students to have sufficient confidence in English learning and at the same time, feel a strong sense of self-achievement. As a result, they will improve their enthusiasm and reduce their negative feelings in the process of English learning, and learning efficiency will be significantly improved.

## Conclusion

In the study, junior high school students are taken as the research subjects to creatively analyze the relationship among students’ psychological needs, teachers’ autonomy support, and English learning effectiveness and English learning engagement. It is found that if students get more psychological needs and perceive more teachers’ autonomy support, their English learning is more effective, and they show higher learning engagement. This research also has certain shortcomings. For example, the research subjects are from the same middle school, and whether the results are true of students from other regions remains to be verified. The research subjects are only junior high school students, and elementary school students or high school students are not involved. In the follow-up, the selection of subjects should be more reasonable.

## Data Availability Statement

The raw data supporting the conclusions of this article will be made available by the authors, without undue reservation.

## Ethics Statement

The studies involving human participants were reviewed and approved by the Northwest University of Political Science and Law Ethics Committee. The patients/participants provided their written informed consent to participate in this study. Written informed consent was obtained from the individual(s) for the publication of any potentially identifiable images or data included in this article.

## Author Contributions

The author confirms being the sole contributor of this work and has approved it for publication.

### Conflict of Interest

The author declares that the research was conducted in the absence of any commercial or financial relationships that could be construed as a potential conflict of interest.
